# Targets' Social Relationships as Antecedents and Consequences of Workplace Bullying: A Social Network Perspective

**DOI:** 10.3389/fpsyg.2019.03077

**Published:** 2020-01-31

**Authors:** Birgit Pauksztat, Denise Salin

**Affiliations:** ^1^Department of Business Studies, Uppsala University, Uppsala, Sweden; ^2^Department of Management & Organization, Hanken School of Economics, Helsinki, Finland

**Keywords:** workplace bullying, social networks, friendship, negative relationships, centrality, social rejection, stochastic actor-oriented modeling

## Abstract

Research on workplace bullying has largely focused on individual and organizational factors that place individuals in a vulnerable position. Although theorists have highlighted social aspects of workplace bullying and its antecedents, the role of individuals' social relations with other members of their organization has rarely been examined empirically. Drawing on insights from social network research and research on social rejection, the purpose of this study was to examine the relationship between exposure to bullying and employees' informal social relationships (here: friendships; negative relationships) with other members of their organization. Data from two waves of surveys among 249 employees in eight organizations in Finland were analyzed using stochastic actor-oriented modeling. We found that employees' centrality (i.e., the number of their relationships) had no effect on exposure to bullying. However, exposure to bullying affected targets' perceptions of their relationships with colleagues: employees who had experienced bullying subsequently reported significantly more friendship relationships, but not significantly more negative relationships, suggesting that aggressive or antisocial responses may be more muted in field settings than in experimental settings. Our study contributes to research on workplace bullying by providing a more detailed understanding of the relationship between workplace bullying and employees' social relations, and by offering insights about the consequences of workplace bullying for targets' social relations.

## Introduction

Although most employees describe their relations with co-workers as neutral or positive (Labianca and Brass, 2006; Venkataramani et al., 2013), some become the target of harmful actions from colleagues or supervisors. Such actions can range from persistent criticism and insulting remarks to physical mistreatment and ostracism. Researchers have studied mistreatment at work using a variety of labels, including bullying, mobbing, harassment, victimization, abusive supervision, and social undermining (Aquino and Thau, 2009; Einarsen et al., 2011; Nielsen and Einarsen, 2018). Here, we use workplace bullying as an umbrella term to refer to “a situation where one or several individuals persistently over a period of time perceive themselves to be on the receiving end of negative action from one or several persons, in a situation where the target of bullying has difficulty in defending him or herself against these actions” (Einarsen and Skogstad, 1996, p. 191).

As a signal of social rejection, exposure to bullying can thwart psychological needs (Trépanier et al., 2016), with severe negative consequences for targets' well-being, work attitudes, absenteeism, and turnover, as well as for organizations (Hauge et al., 2010; Hoel et al., 2011; Nielsen and Einarsen, 2012; Arenas et al., 2015; Nielsen et al., 2019a). Not surprisingly, therefore, numerous studies have sought to identify factors that increase the risk of workplace bullying. To date the focus has largely been on individual dispositions, such as trait negative affect or certain Big Five personality factors, and on aspects of the work environment such as role conflict, work pressure, or high levels of conflict within a team (Salin and Hoel, 2011; Zapf and Einarsen, 2011; Nielsen and Einarsen, 2018; Notelaers et al., 2019a; Zahlquist et al., 2019).

Paradoxically, the role of employees' social relationships has rarely been tested empirically, although theorists have often highlighted social aspects of workplace bullying and its antecedents (Pauksztat and Salin, 2019). As pointed out by Hershcovis and Reich (2013), bullying occurs within a social context, and it is reasonable to assume that this context plays a role in enabling, motivating or precipitating workplace bullying (Salin, 2003; Pauksztat and Salin, 2019). A key aspect of the social context are individuals' informal social relationships with others, such as friendship relations (Homans, 1950; Bridge and Baxter, 1992; Sias, 2009) and negative relationships (Labianca and Brass, 2006). Bullying among school children and adolescents has been found to be strongly linked to social processes (notably status competition; Faris and Felmlee, 2011, 2014) and hence to individuals' friendships and negative relationships (Salmivalli, 2010). Similarly, in the context of workplace bullying, interview studies (Lewis and Orford, 2005; D'Cruz and Noronha, 2011) suggest that social relationships play an important role. However, there is little systematic research on the role of individual employees' social relationships either as antecedents or as consequences of workplace bullying. Social relations appear as a group level variable (e.g., “social community at work;” “social climate”), or in terms of a related concept, “social support” (Skogstad et al., 2011; Van Den Broeck et al., 2011; Francioli et al., 2018). Complicating the picture further, in research within the Job Demands-Resources (JDR) framework (Bakker and Demerouti, 2007; Bakker et al., 2014), social support is often merged into a combined measure of job resources which may also include job characteristics such as autonomy or skill utilization (e.g., Baillien et al., 2011; Balducci et al., 2011). Although these studies suggest that a good social climate and social support can help to protect individuals from becoming targets of bullying, it is difficult to pinpoint the specific contribution of employees' informal social relations in these studies.

The few studies that have examined the association between workplace bullying and informal social relationships suggest that there *is* an association: good relationships had a negative association with the likelihood of bullying behaviors between two individuals, whereas negative relationships had a positive association (Ellwardt et al., 2012; Lyons and Scott, 2012). Moreover, exposure to bullying was positively related to employees' centrality in avoidance networks, and negatively to their centrality in friendship networks (Lamertz and Aquino, 2004; Ellwardt et al., 2012). However, as these studies were based on cross-sectional data, the direction of causality was assumed rather than tested (Hershcovis and Reich, 2013). Indeed, laboratory experiments on social rejection (for an overview, see Richman and Leary, 2009) suggest that the direction of causality could also be the opposite, that is, experiences of social rejection (such as exposure to workplace bullying) may affect interpersonal relationships. To our knowledge, the effects of workplace bullying on targets' social relationships has not yet been explored systematically, although D'Cruz and Noronha's (2011) interview study suggests that targets' efforts to cope with workplace bullying might contribute to “cementing” friendships between targets and their colleagues.

Taken together, existing studies provide some indication that there is an association between workplace bullying and targets' social relationships. However, the nature of this association is not yet well-understood. Thus, there is a need for a more detailed analysis of the effects of employees' social relationships on their exposure to bullying, as well as of the effects of workplace bullying on targets' social relationships.

The purpose of this study is to examine the relationship between workplace bullying and employees' informal social relationships (here: friendships; negative relationships) with other members of their organization. More specifically, the aim of the study is twofold. The first aim is to examine the effects of employees' social relations on their likelihood of being bullied. The second aim is to examine the effects of workplace bullying on targets' friendships and negative relationships. This extends research on social rejection by providing insights into reactions to social rejection (here: workplace bullying) in a field setting. In addition, using stochastic actor-oriented modeling (Snijders et al., 2010) to analyze two waves of panel data on workplace bullying and employees' informal social relations in eight organizations, this study also contributes to a better understanding of the direction of causality.

We begin with an overview of theoretical arguments for considering targets' social relationships as antecedents of workplace bullying and as consequences of bullying, drawing on social network theory and social rejection theory, respectively. We then present the methods and results of our study, and discuss our findings and their implications.

### Targets' Social Relationships as Antecedents of Workplace Bullying

Based on social network theory, employees' informal social relationships with other members of their organization would be expected to affect their likelihood of being bullied. As pointed out by Labianca and Brass (2006), both positive and negative relationships need to be considered in order to obtain a complete picture. Following Labianca and Brass (2006), previous studies have considered networks formed of positive relationships, notably friendship, and networks consisting of negative relationships. Friendship is defined as a voluntary interpersonal relationship characterized by mutual positive affect, trust, and solidarity (Gibbons, 2004; Venkataramani et al., 2013). By contrast, negative relationships are characterized by “an enduring, recurring set of negative judgments, feelings, and behavioral intentions toward another person” (Labianca and Brass, 2006, p. 597). Such negative relationships do not necessarily entail negative *behaviors* (such as bullying or aggression) between two people: “people may have negative encounters without having negative relationships form. Conversely, one person may dislike another person without any observable or latent conflict” (Labianca and Brass, 2006, p. 597). Thus, the extent of the association between negative relations and exposure to negative behaviors such as bullying is an important empirical question.

The effects of individuals' relationships with others can be analyzed at the dyadic level (i.e., a friendship relation between two individuals may affect the likelihood of bullying between those two individuals), or based on their position in the informal social network that consists of the employees in the organization and the relationships or ties amongst them (Borgatti and Halgin, 2011). A key aspect of individuals' position in a network is their centrality, i.e., the extent to which they are engaged in relationships with other individuals (Wasserman and Faust, 1994). Social network researchers have proposed a range of conceptualizations and measures of centrality (e.g., Burt, 1992; Wasserman and Faust, 1994; Borgatti et al., 2002). Here our focus will be on degree centrality, that is, the number of relations that individuals have with other actors in the network (Freeman, 1978/9). More specifically, we will focus on degree centrality based on reciprocated or mutual ties, where both parties agree on the nature of their relationship. This is because reciprocated ties are considered to be stronger than non-reciprocated ties (Mollica et al., 2003; Rivera et al., 2010), and thus should have stronger effects on outcomes such as bullying. We start by considering the effects of centrality in friendship networks on the likelihood of being bullied, and then turn to the effects of centrality in negative networks.

Following social network theory, employees' friendship relationships with other members of their organization should reduce their likelihood of being bullied. Friends are expected to show solidarity, that is, to help and support rather than to undermine or harm each other (Argyle and Henderson, 1984; Bridge and Baxter, 1992; Sias, 2009). Consequently, bullying should be less likely between friends. Having friends is not only advantageous because they are unlikely to bully you, but also because they can provide protection and support against third parties (Brass, 2005; Ibarra et al., 2005; Venkataramani et al., 2013). Moreover, a central position places employees in direct and indirect contact with numerous others, and hence provides them with greater access to and control of resources, such as information, funding, or perhaps an extra pair of hands to help out when needed (Burt, 1992; Brass, 2005). Because negative behavior toward such central employees carries the risk of retaliation not only from themselves (and their informal position of power may place them in an advantageous position to punish the perpetrator), but also from their friends (Ellwardt et al., 2012), their central position in the friendship network is likely to afford them a certain level of protection against becoming targets of bullying. In addition, by interacting with numerous others, they may be able to influence opinions in their favor (Brass, 2005), which further reduces their likelihood of becoming targets of bullying.

From a social network perspective, friendships may thus be considered an important resource. Existing research on workplace bullying supports the notion that resources such as social support may reduce the risk of exposure to workplace bullying (Balducci et al., 2011; Van Den Broeck et al., 2011) and have an additional protective effect when employees face high demands that otherwise increase the risk of bullying (Balducci et al., 2011). These studies are based on broad measures of social support from coworkers and/or supervisors, or combine social support with other types of job resources (such as autonomy or skill utilization). Although this makes it difficult to assess the specific contribution of friendship relations, these studies broadly support the idea that the support provided by friendship relations could contribute to protect individuals from becoming targets of bullying.

In line with this, research on school bullying shows that centrality in the friendship network tends to reduce the likelihood of becoming the target of bullying (Salmivalli, 2010; Sentse et al., 2015). Similarly, in a work context, Ellwardt et al. (2012) found that central employees were less likely to be the target of negative gossip, one form of workplace bullying. Taken together, this suggests that employees' centrality in the friendship network should reduce their likelihood of becoming the target of bullying.

*Hypothesis 1*: Individuals' centrality in the friendship network will reduce their likelihood of exposure to bullying.

Whereas centrality in friendship networks can provide protection from and support against being bullied, centrality in negative networks has been associated with lower access to and control over resources, and lower levels of protection and support (Labianca and Brass, 2006; Venkataramani and Dalal, 2007; Venkataramani et al., 2013). Others might be slower to respond to requests, more circumspect in providing information and other resources, and less willing to go out of their way to help those with whom they have a negative relationship. Moreover, other employees may feel little empathy with those they dislike, and may be reluctant to come to their defense when they are targeted, support them in coping, or help them to bring their case up with managers or others who might be able to intervene. Consequently, bullying someone who does not have the resources to defend him- or herself, and who will not be defended by others may be less risky for perpetrators. In this way, negative relationships could deprive an individual of collegial support and protection, and make him or her an “easy target” (cf. Baillien et al., 2011).

Further, while employees who are central in the friendship network may be able to sway others' opinions in their favor, employees who are central in the negative relationships network may find it difficult to stem the tide of opinion against them. They may be perceived as “not fitting in,” and bullying behaviors such as criticism, gossip or social exclusion may be considered legitimate sanctions for their “inappropriate” opinions, attitudes or behaviors (Ellwardt et al., 2012).

Moreover, negative relationships can be associated with interpersonal conflict (Labianca and Brass, 2006), which, if not managed carefully, can escalate into bullying (Hauge et al., 2007; Einarsen et al., 2011). Inefficient coping with the frustration and stress associated with negative relationships and interpersonal conflicts, as well as heightened suspicion of others (Labianca et al., 1998) can lead individuals to reduce their work effort, complain, or withdraw from social interaction. Such norm-violating behaviors, in turn, can elicit bullying behaviors from colleagues and superiors as retaliatory actions intended to punish the deviating individual (Baillien et al., 2009).

Therefore, we expect that centrality in the negative network will increase an individual's likelihood of becoming a target of bullying.

*Hypothesis 2*: Individuals' centrality in the network of negative relationships will increase their likelihood of exposure to bullying.

In sum, existing research adopting a social network perspective suggests that employees' centrality could be an antecedent of workplace bullying. It suggests that employees' centrality in the friendship network might reduce their exposure to bullying, whereas their centrality in the network of negative ties might increase their risk of being bullied at work. By contrast, as we will discuss in the following section, research on social rejection suggests that employees' friendships and negative relations change *in reaction to* bullying; that is, targets' social relationships may be affected in *consequence* of bullying.

### Effects of Workplace Bullying on Targets' Social Relationships

Explanations of the consequences of workplace bullying have typically been framed in terms of reactions to stressors or traumatic experiences (Høgh et al., 2011; Nielsen and Einarsen, 2012). However, being bullied can also be considered a signal of social rejection, as it involves “negative reactions from other people” that “have the potential to lower people's perceived relational value—the degree to which they believe that others value having relationships with them” (Richman and Leary, 2009, p. 366). Hence, theories concerning effects of social rejection should also help to understand targets' reactions to workplace bullying.

Previous research suggests that social rejection thwarts fundamental psychological needs, notably individuals' sense of social identity and belonging, social status and power (Richman and Leary, 2009; Wesselmann et al., 2015). Findings by Trépanier et al. (2016) suggest that this also applies to bullying. Existing theoretical and empirical studies, largely based on laboratory experiments, suggest three key interpersonal strategies that individuals use for coping with rejection: strengthening social relationships, aggressive behavior, and withdrawal (Richman and Leary, 2009; Wesselmann et al., 2015).

In their efforts to cope with social rejection, individuals who feel rejected may try to strengthen their social relationships, both with the perpetrator and with others who can provide acceptance and support, in order to re-establish their sense of social identity and belonging (Richman and Leary, 2009). In laboratory studies, participants who felt rejected responded with increased efforts to strengthen existing relationships (Derfler-Rozin et al., 2010; Bayer et al., 2019) and showed increased motivation for forming new relationships (Maner et al., 2007).

Alternatively, the need to restore control may take precedence in shaping individuals' coping strategy (Gerber and Wheeler, 2009; DeWall and Bushman, 2011). Aggressive behavior, whether as a defensive or retaliatory response to the perpetrator and/or directed at others, is a way of demonstrating power and re-establishing control (Wesselmann et al., 2015). In laboratory studies, participants who felt rejected showed higher levels of aggression toward the perpetrator or other individuals (Twenge et al., 2001; DeWall et al., 2010; Rajchert and Winiewski, 2017; Rajchert et al., 2019).

Finally, individuals who feel rejected may seek to cope by disengaging and withdrawing from interactions with the perpetrator and/or other people physically, mentally and/or socially (Richman and Leary, 2009). In laboratory studies, being rejected led to an increase in “prevention focused responses” such as withdrawal in order to avoid the reoccurrence of a hurtful situation (Molden et al., 2009).

Extrapolating from the evidence provided by laboratory studies, we might expect three possible coping strategies in reaction to being bullied, which will affect targets' social relationships in different ways. In parallel with research on social rejection, which focused on targets' perceptions, attitudes and behaviors in reaction to social rejection, we are interested in how being bullied affects targets' own perceptions of their relationships with others at their workplace. First, targets of workplace bullying might try to cope by investing in friendships, reflected in a higher number of friendships reported by targets of bullying. Second, they might react with aggressive behavior, thereby increasing their number of negative relationships. And third, they might withdraw from both friendships and negative relationships, thus reporting fewer relationships in both of these networks.

However, it is possible that the effects of social rejection could be different in the context of field settings with ongoing social interactions. First, it is unclear whether the coping strategies identified in laboratory studies immediately after the rejection experience will persist long enough to affect survey responses. Moreover, it is possible that the initial coping strategies identified in laboratory studies will be different in nature from the perhaps more long-term coping strategies that might be found in a field setting. Second, due to the constraints set by the organizational context, such as task interdependence or organizational norms, the need for future collaboration encourages at least a minimum level of professional interaction (Labianca and Brass, 2006). This means for instance that aggressive reactions might be more muted than in laboratory settings, and perhaps less likely to be permitted to escalate and develop into persisting negative relationships. Finally, in a field setting, exposure to bullying occurs in a social context where targets have different relationships with different colleagues, and the nature of these relationships may shape their strategies for coping with bullying. For instance, a target may seek to strengthen his or her relationships with *existing* friends, but may be hesitant to establish *new* friendship ties. In other words, exposure to bullying might have different effects on the maintenance of existing ties and on the creation of new ties. This leads to the following research question:

*Research Question*: What are the effects of exposure to bullying on targets' perceptions of their friendship relationships and their negative relationships?

## Materials and Methods

### Sample

To test the hypotheses and address the research question, we analyse two waves of data from a panel survey in eight public and private sector organizations in Finland: five schools, an IT company, and two cargo ships. The organizations were selected from different sectors to ensure variation. Additional selection criteria were organization size (some 20–45 employees, to enable the collection of whole network data), and working language (Swedish or English, to facilitate communication).

All the organizations' employees at the time of the survey, either on permanent contracts or long fixed-term contracts, were invited to participate in the survey. In total, 249 individuals were employed in the organizations at Time 1 and/or Time 2; 229 individuals were employed at both time points. In total, 237 individuals responded to the survey in at least one wave. Two hundred and twenty-six (of 240) employees responded at Time 1 (response rate: 94%), and 216 (of 238) employees responded at Time 2 (response rate: 91%); 205 employees responded in both waves.

At Time 1, 53.1% of the respondents were women. Respondents' average age was 42.2 years (SD = 10.7), average tenure was 8.5 years (SD = 7.4). Most (81.3%) had permanent contracts, 70.4% worked full time, and 33.3% occupied a managerial position. Respondent characteristics at Time 2 did not differ significantly from those at Time 1. Compared to non-respondents, respondents at Time 1 were more likely to work full time [M = 0.70, SD = 0.46; non-respondents: M = 0.25, SD = 0.45, *t*(236) = 3.35, *p* < 0.01], and to occupy a higher hierarchical position [M = 0.33, SD = 0.81; non-respondents: M = 0.08, SD = 0.29; *t*(236) = 2.46, *p* < 0.05]. There were no significant differences between respondents and non-respondents at Time 2. The organizations differed considerably regarding respondents' demographic characteristics. One-way ANOVAs showed significant differences regarding the percentage of women, full-time employees and managerial employees (*p* < 0.001) in both waves, and significant differences in age (*p* < 0.05) at Time 2.

### Procedure

The study was conducted in accordance with APA ethical standards and the Declaration of Helsinki. Employees' participation in the study was voluntary, and confidentiality was guaranteed. Individual participants were informed about the study in writing and through presentations by the first author. This included information about the way in which the data would be collected, analyzed and reported; in this way, participation reflected “truly informed consent” (Borgatti and Molina, 2005).

Two waves of data, with about 10 weeks between waves, were collected using paper-and-pencil questionnaires. Questionnaires were available in Swedish, Finnish or English, i.e., the respondents' first language or the organization's working language. The questionnaire was prepared in English and Swedish by the first author and a native speaker of Swedish, and translated into Finnish following Brislin (1970). Pilot tests ensured that questions were clear and easy to answer.

### Dependent Variables

Information on the dependent variables was collected in both waves.

#### Exposure to Bullying

This was measured using the Short Negative Acts Questionnaire (Notelaers et al., 2019b; see also Nielsen and Einarsen, 2018; Escartin et al., 2019). Respondents were asked to indicate how often they had been subject to nine types of negative behavior from their colleagues during the last 2 months, such as “insulting or offensive remarks about you as a person, your attitudes or your private life.” Answer categories ranged from 1 (*never*) to 5 (*daily*). Cronbach's alpha was 0.87 at Time 1 and at Time 2. The scale was formed by taking the mean of each respondent's responses. Because the analysis required integer values, values of 1 were coded “1,” values > 1 and ≤ 1.5 were coded “2,” values > 1.5 and ≤ 2 were coded “3,” and values > 2 were coded “4.” The choice of these intervals was based on the distribution of the original variable, which showed a marked clustering at the lower end of the scale (Time 1: M = 1.26, SD = 0.38; Time 2: M = 1.25, SD = 0.40). The correlations between the original and the recoded variables were *r* = 0.89 (*p* < 0.001) at Time 1, and *r* = 0.85 (*p* < 0.001) at Time 2.

#### Friendship Network and Negative Relationships Network

Respondents were given a list of all employees in their organization, and asked to rate the quality of their relationship with each. Answer categories were 1 (*very difficult*), 2 (*difficult*), 3 (*somewhat difficult*), 4 (*neutral*), 5 (*good relationship*), 6 (*friend*), and 7 (*good friend*). The measure was based on the classic network measure of relationship quality by Labianca et al. (1998). Following recommendations by Labianca and Brass (2006), we expanded the scale to a 7-point format to lower the threshold for reporting negative ties, and (in response to respondents' requests) to provide more nuanced categories at the positive end of the scale. Although this does not allow us to consider ambiguous relationships as a separate category (Methot et al., 2017), the measure was suitable for the purpose of our study, i.e., to examine the effects of relations at the positive and at the negative end of the relationship quality continuum. Moreover, empirical studies found strong negative correlations between positive and negative relationships when they had been measured separately (e.g., Huitsing et al., 2012a; Rambaran et al., 2015), thus providing support for measuring them as two poles of a continuum. Using a single question also kept the length of the questionnaire within reasonable limits, thus preventing respondent fatigue and “careless” responses and/or attrition (Marsden, 2011; Ellwardt et al., 2012).

To construct the *friendship network*, a tie was coded “1” when the relationship was described as “friend” or “good friend;” otherwise the tie was coded “0.” For the *negative relationships* network, a tie was coded “1” when the relationship was rated “somewhat difficult,” “difficult,” or “very difficult;” otherwise the tie was coded “0.”

### Organizational Structure and Demographics

Information about organizational structure was obtained from documents or from interviews with managers in each organization.

#### Hierarchical Level

Hierarchical level was coded “0” for employees without supervisory roles, “1” for team leaders and foremen, “2” for those at the next higher level, and “3” for those at the highest hierarchical level.

#### Collaboration Network

Information on collaboration came from organization charts and interviews about team membership, typical patterns of collaboration, and participation in different types of meetings in the organization. From this, we created a dyadic variable coded “1” for pairs of employees whose jobs required close collaboration, and “0” otherwise.

#### Degree Centrality in the Collaboration Network

Based on the collaboration network, degree centrality was calculated as the number of close collaborators, standardized by the number of employees (not counting the focal individual) in their organization.

#### Demographics

Information on demographics was obtained from the organizations or in the survey. This included gender (0 = man, 1 = woman), age (in years), tenure in the organization (in years), and hours per week (0 = part time, 1 = full time).

### Additional Control Variables

Following previous studies pointing to the impact of stressful work environments on bullying (Baillien et al., 2011; Salin and Hoel, 2011) and of the level of bullying in the organization on individuals' reactions to bullying (Huitsing et al., 2012b), we controlled for individuals' perceptions of work pressure, the mean level of work pressure and the mean level of bullying in the organization.

#### Mean Level of Bullying in Organization

This was calculated from individuals' perceptions of exposure to bullying, described above. Group-level reliability was assessed by examining intra-class correlation coefficients [T1: ICC(2) = 0.67, T2: ICC(2) = 0.84].

#### Individual's Perception of Work Pressure

Respondents rated on a seven-point scale how satisfied they were with the work pressure in the organization; answers were reverse-coded so that high values reflected high work pressure.

#### Change in Individual's Perception of Work Pressure

This was calculated by subtracting the Time 1 values from the Time 2 values of individuals' perceptions of work pressure.

#### Mean Level of Work Pressure in the Organization

Respondents were asked to what extent they had noticed problems regarding work pressure in their organization, with answer categories from 1 (*not at all*) to 4 (*very much*). The mean of respondents' scores provided the level of work pressure in each organization. Group-level reliability was assessed by examining intra-class correlation coefficients [T1: ICC(2) = 0.77, T2: ICC(2) = 0.64].

#### Change in Work Pressure in Organization

This was calculated by subtracting the Time 1 values from the Time 2 values of mean level of work pressure in the organization.

### Analytical Approach

We used stochastic actor-oriented modeling with unconditional Method of Moments estimation, implemented in RSiena, version 1.2-12 (Snijders et al., 2010; Ripley et al., 2018). This enabled us to examine factors affecting tie probabilities in co-evolving networks (friendship, negative relationships) and changes in dependent attributes (exposure to bullying), while controlling for endogenous network dynamics and exogenous variables. Details of the method and the model parameters mentioned below are provided by Ripley et al. (2018).

We analyzed the data from all organizations jointly, including all individuals employed in at least one wave (*n* = 249). Although a multilevel approach or a meta-analysis might have been preferable, in practice this was not possible, because separate analyses for each organization and analyses using the multigroup option in RSiena did not converge and/or led to divergent parameter and standard error estimates. We used the programme's default settings, but increased the number of phase 3 iterations to 30,000 to get more reliable standard error estimates. Employees joining or leaving between waves were indicated with composition change files.

We built our model in several steps. First, in line with standard practice we modeled the endogenous dynamics of each network using the default parameters for co-evolving networks [“rate,” “outdegree (density),” “reciprocity”] and dependent attributes (“rate,” “linear shape,” “quadratic shape”). We also tested a range of parameters to model degree distribution (“indegree popularity (sqrt),” “indegree activity (sqrt),” “outdegree activity (sqrt),” “outdegree popularity (sqrt),” “isolates”), and transitivity (“transitive ties,” “transitive triplets,” “geometrically weighted edgewise shared partners,” “number of actors at distance 2”), as well as dummy variables for the organizations. We selected the combination of parameters that gave the best fit while being as parsimonious as possible.

Second, we added the effects of exogenous control variables assessing employees' position in the organization and interaction opportunities (collaboration, hierarchical level, hours per week), demographic characteristics (gender, age, and tenure), organizational levels of work pressure and (for the friendship and negative relationships networks) organizational levels of bullying.

Third, to test the hypotheses and address our research question, we added effects of respondents' centrality on exposure to bullying, and of exposure to bullying on respondents' perceptions of their friendship and negative relationships. To examine the effects of targets' network centrality on exposure to bullying, we added parameters (“recipDeg”) measuring degree centrality based on reciprocated ties in the friendship network and in the negative relationships network, respectively (labeled “Friendship: degree centrality” and “Negative relationships: degree centrality” in **Table 2**). To examine the effect of bullying on targets' perceptions of their relationships, we included exposure to bullying as a so-called “ego” attribute, both for friendship and negative relationships as dependent networks. This parameter [labeled “exposure to bullying (ego)” in **Table 2**] allows us to assess the effect of respondents' exposure to bullying on their likelihood to act as senders of ties (equivalent to their outdegree centrality). Model 1 (**Table 2**) shows the results of analyses assessing the overall effect (known as “evaluation” effect) of exposure to bullying on friendship relations and negative relations.

We conducted additional analyses to explore the likelihood of maintaining existing relationships (“maintenance” effect) and creating new relationships (“creation” effect) separately (Models 2 and 3).

Convergence was indicated by overall maximum convergence ratios below 0.2, and convergence *t*-statistics below 0.1 for all parameters. Further analyses (Lospinoso, 2012; Ripley et al., 2018) suggested that the models presented in **Table 2** had a good fit to the data, indicated by non-significant Monte Carlo Mahalanobis distance tests for auxiliary statistics (indegree distribution; outdegree distribution; geodesic distribution; triad census; behavior distribution).

## Results

### Control Variables

Table 1 shows the means, standard deviations and correlations among the study variables. **Table 2** shows the results of the analyses using stochastic actor-oriented modeling. Regarding our control variables, we found that high levels of work pressure and an increase in work pressure over the study period increased the likelihood of bullying; interestingly, this was largely due to the mean level of work pressure in the *organization*, rather than to respondents' *own* work pressure. Friendship relations were more likely between individuals who collaborated closely, and who were similar in age and tenure. Individuals at higher hierarchical levels and those who experienced high work pressure tended to report fewer friendship relations. Negative relationships were more likely between employees who were similar with regard to work hours and gender, and dissimilar with regard to tenure. Individuals experiencing an increase in their work pressure were more likely to report negative relationships. Women were less likely to report, but more likely to be nominated as targets of negative relationships.

**Table 1:** Means, standard deviations, and bivariate correlations among study variables.

		**M**	**SD**	**1**	**2**	**3**	**4**	**5**	**6**	**7**	**8**	**9**	**10**	**11**	**12**	**13**	**14**	**15**	**16**
1	T1 Exposure to bullying	1.81	0.82																
2	T2 Exposure to bullying	1.81	0.79	0.57^***^															
3	T1 Friendship: degree centrality	2.33	2.21	0.07	0.03														
4	T2 Friendship: degree centrality	2.37	2.25	0.12	0.11	0.76^***^													
5	T1 Negative relationships: degree centrality	0.10	0.40	0.15^*^	0.17^*^	0.10	0.11												
6	T2 Negative relationships: degree centrality	0.15	0.58	0.22^**^	0.23^**^	0.08	0.06	0.73^***^											
7	Gender^a^	0.51	0.50	−0.10	−0.12	0.24^***^	0.17^*^	0.08	0.11										
8	Age	42.04	10.95	−0.10	−0.10	0.04	0.08	0.09	0.10	0.11									
9	Tenure	8.41	7.53	−0.08	−0.10	0.13	0.15^*^	0.08	0.08	−0.04	0.55^***^								
10	Hours per week^b^	0.70	0.46	0.24^***^	0.26^***^	0.10	0.10	0.14^*^	0.16^*^	−0.30^***^	−0.05	0.01							
11	Hierarchical level	0.32	0.80	0.06	0.11	−0.03	0.02	0.08	0.10	−0.20^**^	0.15^*^	0.11	0.27^***^						
12	T1 Collaboration: degree centrality	12.97	10.41	0.13^*^	0.12	−0.02	0.06	0.07	0.03	−0.24^***^	0.00	0.07	0.48^***^	0.43^***^					
13	T1 Mean level of bullying in organization	1.81	0.25	0.30^***^	0.37^***^	0.13^*^	0.13	0.00	−0.04	−0.31^***^	−0.19^**^	0.04	0.32^***^	0.18^**^	0.19^**^				
14	T1 Individual perception of work pressure	3.58	1.38	0.24^***^	0.21^**^	−0.21^**^	−0.12	0.01	0.05	−0.24^***^	−0.09	−0.03	0.14^*^	0.04	0.17^**^	0.14^*^			
15	Change in individual perception of work pressure	−0.03	1.18	0.03	0.12	0.15^*^	0.07	0.03	−0.01	0.05	−0.04	0.02	0.06	0.10	−0.02	0.13	−0.46^***^		
16	T1 Mean level of work pressure in organization	2.04	0.47	0.10	0.20^**^	0.01	0.15^*^	0.05	0.04	−0.26^***^	0.01	−0.08	0.24^***^	0.07	0.22^**^	0.32^***^	0.30^***^	−0.15^*^	
17	Change in work pressure in organization	0.02	0.22	0.13	0.14^*^	0.09	−0.07	−0.04	−0.05	−0.10	−0.18^**^	0.08	0.10	0.11	−0.10	0.45^***^	−0.16^*^	0.25^**^	−0.59^***^

*Based on data from respondents who participated in one or both waves of the survey (n = 237); correlations are calculated using pairwise deletion*.

a *Coded 0 = man, 1 = woman*.

b *Coded 0 = part time, 1 = full time*.

* p < 0.05;

** p < 0.01;

*** *p < 0.001*.

**Table 2:** Stochastic actor-oriented modeling: results.

	**Model 1**		**Model 2**		**Model 3**	
	**Estimate**	**S.E**.	**Estimate**	**S.E**.	**Estimate**	**S.E**.
**DEPENDENT VARIABLE: FRIENDSHIP NETWORK**						
**Structural parameters**						
Rate parameter	4.371	0.250	4.362	0.249	4.391	0.255
Outdegree (density)	−1.495	0.295	−1.511	0.305	−1.483	0.296
Reciprocity	1.753^***^	0.191	1.757^***^	0.189	1.755^***^	0.197
Number of actors at distance 2	0.114^*^	0.051	0.112^*^	0.053	0.116^*^	0.053
Geometrically weighted edgewise shared partners	1.661^***^	0.217	1.655^***^	0.223	1.671^***^	0.225
Indegree popularity (sqrt)	−0.075	0.108	−0.070	0.111	−0.083	0.107
Outdegree popularity (sqrt)	−0.634^***^	0.175	−0.628^***^	0.178	−0.639^***^	0.180
**Exogenous control variables**						
Mean level of bullying in organization	0.025	0.447	−0.007	0.458	0.053	0.437
Individual's perception of work pressure	−0.129^*^	0.053	−0.136^*^	0.056	−0.121^*^	0.054
Change in individual's perception of work pressure	−0.014	0.054	−0.019	0.054	−0.010	0.053
Mean level of work pressure in organization	0.327	0.333	0.318	0.333	0.338	0.326
Change in work pressure in organization	1.076	0.725	1.070	0.729	1.104	0.715
Dummy organization 1	−0.526^**^	0.202	−0.542^**^	0.207	−0.510^*^	0.203
Dummy organization 2	−1.062^***^	0.200	−1.082^***^	0.206	−1.043^***^	0.197
Collaboration (dyadic)	0.442^***^	0.123	0.445^***^	0.124	0.447^***^	0.124
Hierarchical level (alter)	−0.081	0.085	−0.081	0.087	−0.080	0.085
Hierarchical level (ego)	−0.255^**^	0.094	−0.258^**^	0.094	−0.251^**^	0.092
Hierarchical level (same)	−0.073	0.198	−0.075	0.201	−0.072	0.195
Gender^a^ (alter)	0.163	0.126	0.160	0.126	0.166	0.125
Gender (ego)	−0.249	0.144	−0.254	0.144	−0.246	0.143
Gender (same)	0.116	0.111	0.116	0.114	0.117	0.113
Age (alter)	0.002	0.006	0.002	0.006	0.002	0.006
Age (ego)	0.002	0.006	0.001	0.007	0.002	0.007
Age (similarity)	0.601^*^	0.292	0.612^*^	0.296	0.607^*^	0.296
Tenure (alter)	0.012	0.008	0.012	0.008	0.011	0.008
Tenure (ego)	−0.014	0.010	−0.013	0.010	−0.015	0.010
Tenure (similarity)	1.081^**^	0.382	1.085^**^	0.380	1.076^**^	0.379
Hours per week^b^ (alter)	0.087	0.120	0.089	0.120	0.090	0.121
Hours per week (ego)	−0.246	0.132	−0.256	0.137	−0.233	0.134
Hours per week (same)	−0.064	0.109	−0.065	0.111	−0.067	0.109
**Exposure to bullying**						
Exposure to bullying (ego), evaluation	0.289^*^	0.123				
Exposure to bullying (ego), maintenance			0.693^*^	0.345		
Exposure to bullying (ego), creation					0.455	0.304
**DEPENDENT VARIABLE: NEGATIVE RELATIONSHIPS NETWORK**						
**Structural parameters**						
Rate parameter	2.064	0.272	2.050	0.275	2.031	0.264
Outdegree (density)	−7.584	1.034	−7.640	1.020	−7.571	1.050
Reciprocity	1.400^***^	0.417	1.432^***^	0.411	1.411^***^	0.425
Indegree popularity (sqrt)	1.850^***^	0.218	1.827^***^	0.213	1.870^***^	0.225
Outdegree activity (sqrt)	0.833^**^	0.258	0.923^***^	0.237	0.807^**^	0.268
Isolate	2.129^**^	0.752	2.156^**^	0.715	2.194^**^	0.804
**Exogenous control variables**						
Mean level of bullying in organization	−0.483	1.190	0.156	1.035	−0.756	1.293
Individual's perception of work pressure	0.182	0.126	0.209	0.125	0.176	0.129
Change in individual's perception of work pressure	0.504^**^	0.187	0.491^**^	0.186	0.518^**^	0.198
Mean level of work pressure in organization	−0.777	0.772	−0.748	0.745	−0.762	0.790
Change in work pressure in organization	−1.778	1.486	−1.769	1.392	−1.745	1.526
Dummy organization 1	1.157^*^	0.562	1.247^*^	0.557	1.120	0.580
Collaboration (dyadic)	−0.135	0.313	−0.127	0.317	−0.133	0.318
Hierarchical level (alter)	0.097	0.174	0.095	0.173	0.100	0.177
Hierarchical level (ego)	0.157	0.222	0.110	0.216	0.167	0.232
Hierarchical level (same)	−0.508	0.459	−0.526	0.451	−0.524	0.466
Gender^a^ (alter)	1.031^*^	0.461	1.115^*^	0.481	1.047^*^	0.471
Gender (ego)	−1.271^*^	0.503	−1.297^*^	0.503	−1.310^*^	0.515
Gender (same)	0.980^*^	0.440	1.038^*^	0.453	0.981^*^	0.453
Age (alter)	−0.001	0.013	−0.001	0.013	−0.001	0.013
Age (ego)	−0.002	0.020	0.000	0.018	−0.003	0.020
Age (similarity)	−0.063	0.728	0.082	0.719	−0.054	0.745
Tenure (alter)	−0.006	0.020	−0.003	0.020	−0.008	0.021
Tenure (ego)	−0.011	0.029	−0.024	0.027	−0.007	0.030
Tenure (similarity)	−1.890^*^	0.929	−1.810^*^	0.913	−2.012^*^	0.946
Hours per week^b^ (alter)	−0.372	0.350	−0.405	0.348	−0.385	0.352
Hours per week (ego)	0.404	0.447	0.555	0.437	0.287	0.452
Hours per week (same)	0.790^*^	0.345	0.792^*^	0.346	0.800^*^	0.346
**Exposure to bullying**						
Exposure to bullying (ego), evaluation	0.512	0.307				
Exposure to bullying (ego), maintenance			−0.031	0.730		
Exposure to bullying (ego), creation					1.196	0.646
**DEPENDENT VARIABLE: EXPOSURE TO BULLYING**						
**Structural parameters**						
Rate parameter	1.313	0.202	1.308	0.204	1.316	0.202
Linear shape	−0.101	0.304	−0.095	0.300	−0.100	0.300
Quadratic shape	−0.774^**^	0.277	−0.760^**^	0.271	−0.765^**^	0.276
**Exogenous control variables**						
Individual's perception of work pressure	0.261	0.150	0.256	0.151	0.256	0.150
Change in individual's perception of work pressure	0.352	0.180	0.349	0.180	0.350^*^	0.178
Mean level of work pressure in organization	2.820^**^	0.954	2.825^**^	0.949	2.814^**^	0.957
Change in work pressure in organization	5.096^**^	1.861	5.074^**^	1.834	5.100^**^	1.859
Dummy organization 1	1.309^*^	0.592	1.312^*^	0.591	1.312^*^	0.597
Collaboration (degree centrality)	0.003	0.018	0.002	0.018	0.003	0.018
Hierarchical level	0.064	0.218	0.061	0.217	0.056	0.217
Gender^a^	0.511	0.422	0.510	0.412	0.520	0.413
Age	0.010	0.020	0.009	0.020	0.010	0.020
Tenure	−0.039	0.030	−0.037	0.029	−0.038	0.030
Hours per week^b^	0.178	0.408	0.170	0.404	0.181	0.408
**Network effects**						
Friendship: degree centrality	−0.061	0.095	−0.062	0.093	−0.063	0.096
Negative relationships: degree centrality	0.906	0.624	0.903	0.609	0.879	0.607

* p < 0.05;

** p < 0.01;

*** *p < 0.001*.

a *Coded 0 = man, 1 = woman*.

b *Coded 0 = part time, 1 = full time. For networks as dependent variables, demographic attribute variables are included as characteristics of individuals acting as senders of ties (“ego” attributes), recipients of ties (“alter” attributes), and sender-recipient pairs (“similarity” or, for dichotomous variables, “same”)*.

### Effects of Targets' Social Relationships on Workplace Bullying

According to Hypothesis 1, individuals' centrality in the friendship network should have a negative effect on subsequent exposure to bullying behaviors. As shown in Table 2, the effect was in the expected direction, but non-significant (Model 1: estimate = −0.061, s.e. = 0.095, *n.s*.). Thus, Hypothesis 1 was not supported. To assess the robustness of this result, we conducted additional analyses replacing degree centrality with indegree centrality (i.e., the number of nominations received by an individual), with outdegree centrality (i.e., the number of nominations made by an individual), or including indegree and outdegree centrality simultaneously. We also tested interactions between indegree and outdegree centrality. Moreover, we reran the analyses using the natural logarithm of the centrality variables (calculated as the base-e logarithm of centrality plus one). However, none of the effects were significant.

Hypothesis 2 predicted a positive effect of centrality in the network of negative relationships on exposure to bullying. However, although positive as expected, the effect was non-significant (Model 1, estimate = 0.906, s.e. = 0.624, *n.s*.). This provided no support for Hypothesis 2. As for Hypothesis 1, we assessed the robustness of this result by conducting additional analyses, in which we replaced degree centrality with indegree centrality, outdegree centrality, or both indegree and outdegree centrality. We also tested interactions between indegree and outdegree centrality, as well as using the natural logarithm of the centrality variables. However, none of the effects were significant^1^.

### Effects of Workplace Bullying on Targets' Social Relationships

Concerning the research question about the effects of workplace bullying on targets' perceptions of their relationships with others, we found a significant positive effect of exposure to bullying on friendship (Model 1, evaluation effect: estimate = 0.289, s.e. = 0.123, *p* < 0.05), that is, targets of bullying reported significantly more friendship ties in the following wave than other employees. Additional analyses showed that those exposed to bullying were especially likely to maintain existing friendships (Model 2, maintenance effect: estimate = 0.693, s.e. = 0.345, *p* < 0.05), i.e., targets who perceived a relationship as a “friendship” at Time 1 were more likely to continue to perceive that relationship as a “friendship” at Time 2. The effect of exposure to bullying on the creation of new friendship relations was positive as well, but not significant (Model 3, creation effect: estimate = 0.455, s.e. = 0.304, *n.s*.).

Concerning the effect of exposure to bullying on targets' perceptions of negative relationships, we found that the overall effect was positive but above the 0.05 significance threshold (Table 2, Model 1, evaluation effect: estimate = 0.512, s.e. = 0.307, *p* < 0.1). In other words, there was a tendency for targets to report more negative relationships in the following wave, but this tendency was not significant at the 0.05 level. Additional analyses suggested that for negative ties, the creation effect (Model 3, creation effect: estimate = 1.196, s.e. = 0.646, *p* < 0.1) was stronger than the maintenance effect (Model 2, maintenance effect, estimate = −0.031, s.e. = 0.730, *n.s*.). That is, targets were somewhat (although not significantly) more likely to report new negative ties in the following wave, but exposure to bullying had no effect on the maintenance of negative ties.

Additional analyses showed that the evaluation and creation effects of exposure to bullying on negative relationships were considerably stronger when exogenous control variables were excluded from the model. More specifically, there was a significant positive evaluation effect (estimate: 0.480, s.e. 0.212, *p* < 0.05) and a significant positive creation effect (estimate: 1.008, s.e. 0.422, *p* < 0.05). The maintenance effect remained non-significant (estimate: 0.145, s.e. 0.662, *n.s*.).

As evident from this, our findings provide no evidence of withdrawal, i.e., there was no significant decrease in targets' positive and negative relationships.

## Discussion

This study adopted a social network approach to examine how targets' informal social relationships affect their exposure to bullying, and how exposure to bullying in turn affects their social relationships. Based on two waves of panel data from eight public and private sector organizations in Finland, we found that targets' centrality in the friendship network and in the negative relationships network had no effect on their likelihood of being bullied. However, exposure to bullying affected targets' social relationships: targets were significantly more likely to maintain existing friendships. We will discuss each of these findings in turn.

Employees' social relationships have not traditionally been explored in connection with workplace bullying, although theoretical considerations (Labianca and Brass, 2006) suggest that they might play a role. Research on school bullying (Salmivalli, 2010) and existing cross-sectional studies in a workplace context (Lamertz and Aquino, 2004; Ellwardt et al., 2012; Lyons and Scott, 2012) support this intuition. However, contrary to previous assumptions, we found that exposure to bullying was not predicted by targets' centrality in informal social networks. Instead, our results suggested that bullying was primarily related to the level of work pressure in an organization.

While the strength of the effect of employees' social relationships on exposure to bullying may have been overestimated in previous cross-sectional studies, two possible alternative explanations come to mind. First, the majority of respondents in our study experienced low frequencies of bullying behaviors. Hence, our results may be specific to low-frequency exposure to bullying behaviors. As pointed out by Notelaers (2011), the antecedents of bullying may differ depending on the nature and intensity of the negative behaviors, and it is possible that the protection and support provided by social relationships is most relevant for reducing severe bullying and/or certain forms of bullying. This could explain why earlier studies have found significant effects of network centrality on bullying among schoolchildren (e.g., Salmivalli, 2010; Sentse et al., 2015), which typically involves more direct, physical forms of negative behavior than bullying among adults (Monks et al., 2009).

Second, social relationships might predict school bullying, but not workplace bullying, because the two are driven by different factors. Bullying in schools may be largely driven by children's search for social status and dominance (Salmivalli, 2010; Faris and Felmlee, 2011, 2014), hence children's position in the social structure should be a key antecedent. By contrast, workplace bullying may be largely due to high work pressure that contributes to heightened levels of tension, which find expression in negative interpersonal behavior (Baillien et al., 2009, 2011). This explanation seems to fit well with the findings in our study, and is in line with the work environment hypothesis of bullying, which highlights deficiencies in the work environment as the main cause of workplace bullying (Salin and Hoel, 2011).

Concerning the impact of bullying on targets' social relations, we found that targets tended to report more friendship relationships. In particular, they were more likely to maintain existing friendships than those who did not experience bullying. This is in line with theoretical expectations concerning coping strategies (Richman and Leary, 2009) and empirical findings in laboratory studies that found that individuals were more likely to invest in social relationships following experiences of rejection, in order to affirm their sense of safety and belonging (Richman and Leary, 2009; Wesselmann et al., 2015).

Field studies on workplace bullying, as well as research on friendship development suggest an alternative explanation, namely that friendships may be strengthened as a byproduct of targets' attempts to cope with bullying. Field studies on workplace bullying identified reaching out to colleagues for support as one of the most common forms of coping (Høgh et al., 2011). Due to expectations of mutual solidarity and support between friends (Argyle and Henderson, 1984; Bridge and Baxter, 1992; Sias, 2009), targets of bullying are likely to turn to their friends for support, and friends are likely to provide emotional and/or practical support. As noted by Sias (2009), receiving the expected support confirms that the other can indeed be considered a “true friend.” Together with the shared experience of a crisis situation, this might contribute to strengthening the relationship (Sias, 2009). These findings do not seem limited to European or North American contexts. For instance D'Cruz and Noronha (2011), in their qualitative interview study among call center workers in India, found that being bullied often strengthened the friendship between targets and their friends.

While our results suggest that being exposed to bullying is associated with an increase in reported friendships, we would strongly caution against interpreting this to mean that being exposed to bullying in itself has “positive” outcomes for targets. The strengthening of friendship relations should not be seen as a direct and automatic outcome of being bullied, but may be best interpreted as a byproduct of targets' strategies for coping with bullying. Research on the effects of bullying suggests that social support acts as a moderator that can buffer the negative effects of bullying. For instance, there has been research on how social support may protect against mental distress and sickness absence in cases of bullying (Nielsen et al., 2019b). While this is beyond the scope of this study, it does suggest that holding on to friends who may be able to provide social support (as the employees in our study seemed to do) is a potentially helpful coping strategy.

In experimental studies, social rejection also increased the likelihood of aggressive or antisocial responses (Gerber and Wheeler, 2009). In our data, the direction of the effect was the same as in experimental studies (i.e., those who experienced bullying were somewhat more likely to report negative relationships), but this effect was not significant. Three possible explanations for this weaker effect in our study, compared to previous experimental studies, come to mind. First, while our results consistently suggest that exposure to bullying does not affect the *maintenance* of negative relationships, the lack of significance of the evaluation and creation effects of bullying on targets' negative relationships might be due to the low frequency of bullying and/or negative relationships in our data. Second, following Labianca and Brass (2006), this finding could be due to constraints set by the organizational context, where the need for future collaboration encourages at least a minimum level of professional interaction. Hence the expression of aggressive reactions might be more muted than in laboratory settings, and they may be less likely to be permitted to escalate and develop into persisting negative relationships. Third, it is tempting to speculate that in the context of the present study where exposure to bullying was associated with high work pressure, it could be that employees attribute negative behavior to tensions caused by high work pressure (Baillien et al., 2009, 2011), rather than to the perpetrator's ill-will. This might lead employees to support (rather than blame) each other, thus encouraging the strengthening of friendship ties rather than the intensification of negative ties. Further, although new negative relationships may develop in reaction to a hurtful experience, they may be short-lived, especially when the perpetrator's behavior can be attributed to the situation (e.g., a stressful work environment) rather than to personal dislike of the target. This suggests that the effect of exposure to bullying might depend on the severity of bullying, and whether it can be attributed to the perpetrator's intention (e.g., intention to harm the target) or to the situational context (e.g., frustration caused by high work pressure). Thus targets' reactions to social rejection may not only be shaped by their personal characteristics (e.g., Rajchert and Winiewski, 2017; Weerdmeester and Lange, 2019), but also by their interpretation of the causes and motivation of the perpetrators' behaviors. As pointed out by Huitsing et al. (2012b), this interpretation is influenced by the social context.

The low frequency of exposure to workplace bullying behaviors in our data, and the association between bullying and work pressure may also explain the absence of withdrawal in reaction to bullying in our data. As noted by Richman and Leary (2009, p. 382), withdrawal “may be influenced by the degree to which people interpret the rejection as a reflection of their general relational value or social acceptability.” And although withdrawal has been identified as a coping strategy in cases of intense bullying (Lewis and Orford, 2005), it may be less likely in reaction to behaviors that are attributed to situational circumstances or characteristics of the group or organization (see also Huitsing et al., 2012b).

Finally, our findings provided some indications that exposure to bullying behaviors could have different effects on the maintenance of existing relationships and on the creation of new relationships. This distinction has not received systematic attention to date. Developing and testing theoretical explanations of these different effects will be an important task for future research.

## Limitations

Some limitations of this study should be noted. First, as discussed above, most of the respondents in our study experienced low levels of bullying, restricting the range of our dependent variable. However, it is possible that both the antecedents and consequences of bullying will differ depending on the intensity and perhaps the type of the bullying (Notelaers, 2011). We therefore encourage future research to explore this in more detail, for instance by using a bullying measure with dummy variables for low, medium, and high scores. Unfortunately this was not possible with our data, due to the small number of cases with high scores on the bullying scale.

Similarly, the number of negative relationships was relatively low in the organizations in our study, and, together with the low levels of bullying in the organizations in our study, this may be one possible reason for the non-significant effects of exposure to bullying on negative relationships in particular. Because negative ties tend to be relatively infrequent (between one to eight percent of the total number of ties) in intraorganizational networks (Labianca and Brass, 2006), this is not a problem that is limited to our study, but is likely to apply to future studies as well. Here meta-analyses may be useful to get a better understanding of the relationship between exposure to bullying and negative relations.

Second, we collected social network data for social relations, but not for bullying, for which we used a widely used, validated scale (Nielsen and Einarsen, 2018; Notelaers et al., 2019b; Escartin et al., 2019). In addition to reducing the likelihood of common method bias (Podsakoff et al., 2003), this was appropriate given our research questions about the relationship between exposure to workplace bullying and employees' informal social relationships. However, this did not allow us to compare the effect of bullying on the relationship between targets and perpetrators on the one hand, and targets and non-perpetrators on the other hand. Thus, future studies should also take into account who bullies whom, and consider the characteristics of perpetrators, targets and their relationship with each other (Hershcovis and Reich, 2013). However, this should not jeopardize measurement validity and response rates. Because collecting social network data can be time consuming and tiring for respondents, network measures tend to consist of only one item (Marsden, 2011). However, for a complex phenomenon such as exposure to bullying, one item measures (e.g., presenting respondents with one complex, multi-faceted item; Lyons and Scott, 2012)—can raise questions about validity. Moreover, respondents' hesitations about reporting social ties may be even stronger for highly sensitive behaviors such as bullying, thus increasing the risk of non-response and/or response biases (Marsden, 2011).

Third, our study was based on a two-wave survey in eight Finnish organizations in a variety of settings. Although this enhances generalizability, the small number of organizations and their small size, combined with the sparseness of the negative relationships network, prevented more detailed multilevel analyses of similarities and differences between the organizations. Collecting whole network data from a larger number of organizations in different settings may be difficult in practice. However, for research questions concerning individuals' perceptions of their social environment, collecting personal network data might be a viable alternative (Crossley et al., 2015).

Moreover, although our sample included organizations from different sectors, all of the data were collected in Finland and as such represent one specific cultural and demographic context. For instance, in global comparison, the Nordic countries (which include Finland) have been reported to have lower levels of bullying than many other countries (Nielsen et al., 2010). In addition, whereas in some countries targets typically report being bullied by supervisors, targets in the Nordic countries often report being bullied by peers (Zapf et al., 2011). Furthermore, Finland has historically been relatively homogenous, with little cultural or ethnic diversity, and a relatively high level of gender equality (e.g., World Economic Forum, 2018). All of these contextual factors may have affected the results. For instance, it is possible that the effects of exposure to bullying found in our study (e.g., maintaining existing friendship ties, and perhaps creating new negative ties) might be more pronounced in contexts where group boundaries are strengthened by strong demographic faultlines. We therefore strongly encourage researchers to replicate the study in other contexts, notably in organizations characterized by more heterogeneity (especially regarding social categories such as race) and by higher levels of bullying.

## Conclusion

Contrary to previous assumptions, our findings suggested that targets' informal social relationships did not affect their exposure to bullying; however, workplace bullying affected employees' perceptions of their relationships with other members of their organization. Employees who had experienced bullying subsequently were more likely to report friendship relationships; more specifically, they were more likely to maintain existing friendship relationships. However, in contrast to laboratory studies, exposure to bullying did not lead to a significant increase in employees' negative relationships or withdrawal, suggesting that aggressive or antisocial responses may be more muted in field settings than in experimental settings. Our study contributes to research on workplace bullying by providing insights into the effects of bullying on targets' social relations. Our findings also point to the need for future studies to take into account the intensity and motivation of bullying behaviors, and to further explore the role of social relationships by considering who bullies whom.

## Data Availability Statement

The datasets generated for this study will not be made publicly available in order to ensure confidentiality, which had been guaranteed to participants. Enquiries about the data should be directed to the corresponding author.

## Ethics Statement

Ethical review and approval were not required for this study on human participants in accordance with the local legislation and institutional requirements. Written informed consent from the participants was inferred through the completion of the survey.

## Author Contributions

BP collected and analyzed the data and initiated and wrote the manuscript. DS advised on the use of the S-NAQ scale and its translation in the survey, and contributed to the entire manuscript, especially regarding research on workplace bullying. Both BP and DS read, commented on and approved the final manuscript.

### Conflict of Interest

The authors declare that the research was conducted in the absence of any commercial or financial relationships that could be construed as a potential conflict of interest.

## Footnotes

^1^To assess whether these results were specific to the centrality measure we had selected (i.e., degree centrality), we conducted additional analyses using alternative measures of individuals' centrality in the friendship network (notably betweenness, effective size, efficiency, constraint and ego-network density; for more information on these measures, see Burt, 1992; Borgatti et al., 2002) as predictors of exposure to workplace bullying. However, the effects of these alternative variables on workplace bullying were non-significant even when all other variables except structural parameters were excluded (calculating these alternative measures for the negative relationship networks was not meaningful due to the sparseness of these networks).
